# A Tumor-Like Lump in the Palm Caused by an Inconspicuous-for 75 Years-Bullet

**DOI:** 10.1155/2020/8898016

**Published:** 2020-07-08

**Authors:** Efstratios D. Athanaselis, Apostolos Fyllos, Nikolaos Stefanou, Socrates E. Varitimidis, Dimitrios Giannikas

**Affiliations:** ^1^Department of Orthopaedics & Musculoskeletal Trauma, University General Hospital of Larissa, Biopolis, TK41110 Larissa, Greece; ^2^Le Centre de la Main, Rhéna Clinique de Strasbourg Centre de la Main, 10 Rue François Epailly 67000 Strasbourg, France

## Abstract

**Case:**

An unusual case of a foreign body in the hand is described here. Excision of a tumor-like soft tissue mass revealed a 75-year-old World War II bullet fragment of which patient was unaware.

**Conclusion:**

Differential diagnosis of hand lumps and inflammatory reaction must always include retained foreign bodies even after a very long period of posttraumatic quiescence or patient's inability to provide a relative injury case history.

## 1. Introduction

Hand injuries complicated with foreign bodies are common. Foreign bodies are usually removed within a few hours after the incidence of injury. However, there is a minority of patients who are unaware of intrusion and the foreign body is accidentally detected a long time after. Residual foreign bodies generate both acute and chronic inflammatory reactions. Chronic inflammation leads to creation of granulomatous tissue which isolates the foreign body creating a surrounding capsule.

## 2. Statement of Informed Consent

Patient was informed that data concerning the excision will be submitted for publication and has agreed.

## 3. Case Report

**Figure 1:**
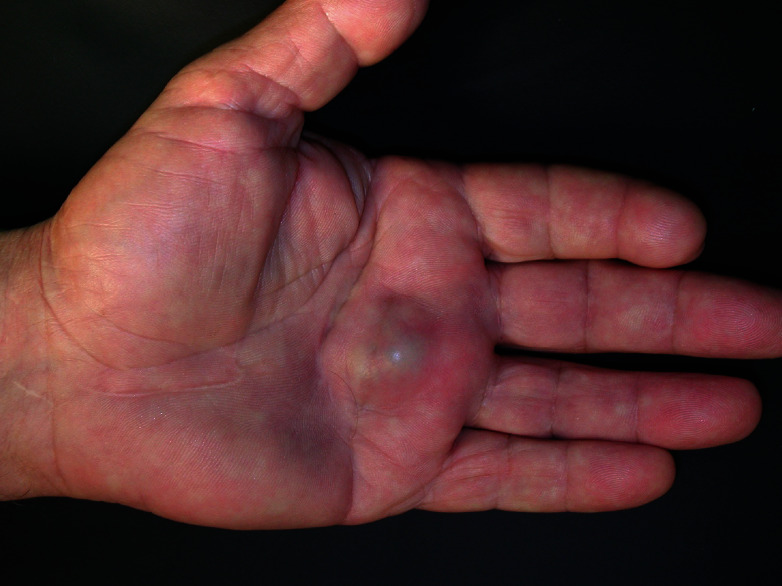
Lump in the region of heads of the 3^rd^ and 4^th^ metacarpal bone. A scar can be noticed proximally, on the palmar skin of the wrist.

An 84-year-old male ex-farmer was admitted to the orthopaedic department due to an inflamed subcutaneous mass in his left palm over the heads of the 3^rd^ and 4^th^ metacarpal bones (Figure 1). The lump was slightly tender on palpation without interfering with finger flexion. Mass was noticed 3 months before without trauma history and kept growing since then.

Routine blood investigation was normal. Plain radiographs revealed a nonradiolucent object 5 × 2 mm in size, located in the region of the mass. A well-defined, avascular, mass was depicted in the MRI.

**Figure 2:**
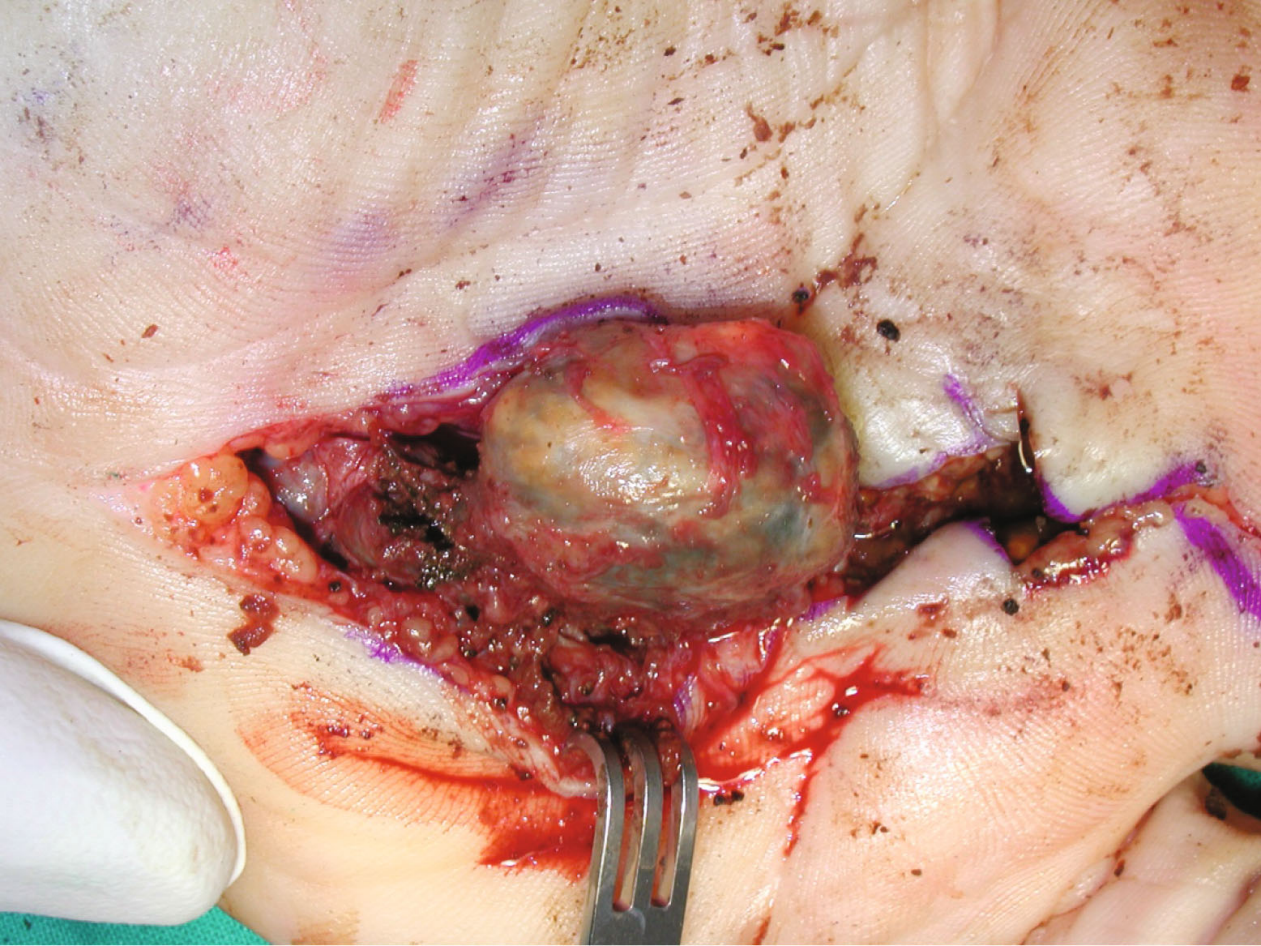
Excision of the cyst.

Patient was scheduled to be operated for an excisional biopsy of a hand soft tissue tumor. Intraoperatively, a thick-wall cyst containing brown-colored fluid, a metallic object, and various particles resembling rusty products, was excised (Figure 2).

After describing our findings to the patient, he recalled a long-forgotten incidence during World War II, 75 years ago: a “superficial” gunshot injury in the palmar aspect of the wrist near its crease. Ricochet of a bullet caused a wound that was healed with no complication, leaving a hardly noticed scar.

Histological examination of the excised mass confirmed our suspicion. The residual foreign body was a bullet which, being deposited subcutaneously in the palm of the hand for more than 70 years, had been subjected to excessive corrosion in the biological environment and progressively isolated in a granulomatous tissue capsule.

The postoperative healing was uneventful. On the follow-up examinations at the 1^st^, 3^rd^, and 6^th^ month, no sign of inflammation or recurrence has been noticed and hand function was unproblematic. Further follow-up was considered unnecessary.

## 4. Discussion

Foreign bodies are a common problem in orthopaedics. The upper limb and particularly the hand are usually involved in accidents (mostly labor) that cause penetrating wounds. Broken parts of metal, wood, and glass may remain in the body after such penetrating injury, often causing pain and worry in a patient. Foreign bodies are usually removed in the emergency department by local anesthesia or in the operating theater under regional (or even general anesthesia in more complex cases). However, there are cases in which an inflammatory reaction, months or years later, brings a patient to a hospital and although the initial injury may be almost forgotten, a remaining foreign body is revealed.

The intensity of the reaction depends on the type of the material, the presence of any natural or synthetic toxins, and the location of the deposition [1, 2]. Metal foreign bodies under corrosion in a biological environment can elicit even generalized reactions due to human body metallosis. Metal components either of one specific metal or alloys (bullets are made of alloys, typically containing antimony, Sb) can be deposited in specific organs affecting their function, inducing Fe, Cu, and Zn dyshomeostasis, and potentially triggering neurodegenerative disorders [3].

In our case, chronic inflammation generated progressively granulomatous tissue and the formed capsule isolated the metallic debris preventing systematic reactions [4]. Synovitis, effusion, and acute onset of pain of a joint can be present in case a foreign body is deposited in or near the joint [5]. However, such granulomas in nearby joints and tendons may lead to decreased range of movement and erythema as well. There also have been reports of bone erosion in the hand, treated unsuccessfully with antibiotics on the basis of a misdiagnosed osteomyelitis, in which after all, resection biopsy revealed granuloma surrounding a sterile foreign body [1].

The diagnosis of a retained foreign body depends on clinical suspicion and is based on the accurate patient's history and imaging studies. Various methods for visualizing different materials have been reported [6]. Plain radiograph taken in multiple projections is the first step even though some foreign bodies are “silent” [7]. Glass or gravel is best seen with soft tissue imaging techniques. Computed tomography may detect plastic, glass, and wood, and MRI remains a more expensive backup for all types of materials, but still, ultrasound sonography remains the “gold standard” in the diagnosis of foreign bodies in the hand due to lower cost among other techniques and no radiation [6, 8]. Examination by soft tissue u/s has high specificity in detecting foreign bodies' presence. In general, X-rays in combination with ultrasonography can reveal and localize almost all foreign bodies in the hand [9].

Removal of the foreign body resolves inflammatory reaction, and usually, no recurrence is noted.

In our case, it is also remarkable that the patient, despite his daily heavy manual activities for decades, had no symptoms at all in the palm for 75 years. Furthermore, it was very difficult for him to correlate the mass formation with an insignificant, previous injury about 10 cm away from its location.

## Data Availability

No data were used to support this study.

## References

[1] Kann S. E., Jacquemin J., Stern P. J. (1997). Simulators of hand infections. *Instructional Course Lectures*.

[2] Ramanathan E. B. S., Luitz C. P. J. (1990). Date palm thorn synovitis. *Journal of Bone and Joint Surgery*.

[3] Andrade V. M., Aschner M., dos Santos A. P. M. (2017). Neurotoxicity of metal mixtures. *Advances in Neurobiology*.

[4] O'Connor C. R., Reginato A. J., DeLong W. G. (1988). Foreign body reactions simulating acute septic arthritis. *The Journal of Rheumatology*.

[5] Reginato A. J., Ferreiro J. L., RiesterO'Connor C. (1990). Clinical and pathologic studies of twenty-six patients with penetrating foreign body injury to the joints, bursae, and tendon sheaths. *Arthritis and Rheumatism*.

[6] Marquis G. P. (1989). Radiolucent foreign bodies in the hand. *The Journal of Trauma*.

[7] Hansson G., Beebe A. C., Carroll N. C., Donaldson J. S. (2009). A piece of wood in the hand diagnosed by ultrasonography. *Acta Orthopaedica Scandinavica*.

[8] Lejeune A., Nizet M. (1993). Detection of foreign bodies in hand. *The Journal of Hand Surgery*.

[9] Bray P. W., Mahoney J. L., Campbell J. P. (1995). Sensitivity and specificity of ultrasound in the diagnosis of foreign bodies in the hand. *The Journal of Hand Surgery*.

